# Post-traumatic stress disorder after drowning 

**Published:** 2022-12

**Authors:** Sima Garshasbi

**Affiliations:** ^*a*^ Department of Health in Emergencies and Disasters, School of Public Health, Tehran University of Medical Sciences, Tehran, Iran.

## Abstract

**Background:**

Post-traumatic stress disorder (PTSD) is a mental disorder that occurs after a traumatic event. Drowning can cause PTSD. This study aimed at highlighting the issue of PTSD in people with drowning experience and witnesses. PTSD is different in people who experience drowning, depending on the age and physical and mental health of the drowned. The experience of drowning in the sea, swimming pool, fishing lake, river, agricultural pond, swamp, etc. can create a variety of post-traumatic stress scenarios for the drowned person, companions, and witnesses of the accident.

**Methods:**

This article is the result of a review of articles about PTSD after drowning extracted from Scopus, Google Scholar, and PubMed databases. The time span for the search was last five years. The author studied the articles and based on the knowledge of health psychology and life experience in the coastal areas of northern Iran, the following results were obtained.

**Results:**

PTSD often changes a person's physical and emotional response in different situations. Extreme fear of water, the feeling of shame, aggression, depression, anxiety disorders, and constant disturbing thoughts are among the most important outcomes of stress after a drowning accident.

**Conclusions:**

Certain solutions are suggested for the prevention, control, and treatment of drowning post-traumatic stress. Teaching swimming skills, knowing one’s self-skills, and strengthening self-control on all occasions are the best strategies for PTSD prevention. Referring to psychiatrists/psychologists is the best solution for people with drowning experiences and drowning witnesses for the treatment of PTSD. Post-traumatic stress disorder can affect the daily life of these people for their whole life. If left untreated, PTSD can cause irreparable physical, psychological, and social effects.

**Keywords:**

PTSD, Post-traumatic stress disorder, Drowning

